# Effects of Plant and Soil Amendment on Remediation Performance and Methane Mitigation in Petroleum-Contaminated Soil

**DOI:** 10.4014/jmb.2006.06023

**Published:** 2020-10-23

**Authors:** Yoonjoo Seo, Kyung-Suk Cho

**Affiliations:** Department of Environmental Science and Engineering, Ewha Womans University, Seoul 03760, Republic of Korea

**Keywords:** Rhizoremediation, petroleum-contaminated soil, chemical nutrient, compost, methane emission

## Abstract

Petroleum-contaminated soil is considered among the most important potential anthropogenic atmospheric methane sources. Additionally, various rhizoremediation factors can affect methane emissions by altering soil ecosystem carbon cycles. Nonetheless, greenhouse gas emissions from soil have not been given due importance as a potentially relevant parameter in rhizoremediation techniques. Therefore, in this study we sought to investigate the effects of different plant and soil amendments on both remediation efficiencies and methane emission characteristics in dieselcontaminated soil. An indoor pot experiment consisting of three plant treatments (control, maize, tall fescue) and two soil amendments (chemical nutrient, compost) was performed for 95 days. Total petroleum hydrocarbon (TPH) removal efficiency, dehydrogenase activity, and *alkB* (*i.e.*, an alkane compound-degrading enzyme) gene abundance were the highest in the tall fescue and maize soil system amended with compost. Compost addition enhanced both the overall remediation efficiencies, as well as *pmoA* (*i.e.*, a methane-oxidizing enzyme) gene abundance in soils. Moreover, the potential methane emission of diesel-contaminated soil was relatively low when maize was introduced to the soil system. After microbial community analysis, various TPH-degrading microorganisms (*Nocardioides, *Marinobacter*, Immitisolibacter, Acinetobacter, Kocuria, Mycobacterium, Pseudomonas, Alcanivorax*) and methane-oxidizing microorganisms (*Methylocapsa, Methylosarcina*) were observed in the rhizosphere soil. The effects of major rhizoremediation factors on soil remediation efficiency and greenhouse gas emissions discussed herein are expected to contribute to the development of sustainable biological remediation technologies in response to global climate change.

## Introduction

Petroleum hydrocarbons (PHs) are the most widely used fossil fuels worldwide and refined PH products (*e.g.*, gasoline, diesel, lubricating oil, etc.) are fundamental components of entire industrial processes. However, PHs are also major environmental contaminants and enter the ecosystem through various ways such as leakage of underground storage tanks, oil spills, transportation, and industrial processes [1].

Soils become extremely hydrophobic when polluted with PHs, thereby causing water deficiencies and an insufficient supply of major nutrients such as nitrogen and phosphorus, which are essential for plant and soil microorganism growth [2]. This leads to a reduction in the diversity and activity of soil biota including plants, animals, and microorganisms, and consequently affects the overall soil ecosystem [3]. Additionally, soil PH pollution impairs not only the specific contaminated area but also the aesthetic and economic value of the soil by causing odor problems associated with volatile PHs [4]. Thus, considering the levels of incidence and potential damage, PH soil contamination is a major environmental problem that needs to be rapidly dealt with through appropriate remediation techniques.

Rhizoremediation, a representative biological remediation technique for treating petroleum-contaminated soils, removes organic pollutants by increasing the metabolic activity of rhizosphere microorganisms through plant synergisms [4]. Various soil amendments are usually added during rhizoremediation to improve remediation efficiency by enhancing soil physicochemical characteristics such as fertility, water content, and nutrients [3, 5]. Particularly, the addition of soil amendments alters the amount of soil carbon and nitrogen utilized by soil microorganisms. For example, the application of organic fertilizers including manure was reported to increase soil methane emissions by supplying nutrients and organic acids [6].

Plants may also become involved in rhizosphere microorganism community composition and recent studies have reported that soil methane emissions were modified by changes in the microbiota composition involved in soil organic matter metabolic processes, which in turn had been altered by plant root exudation [7]. However, the effects of plants on soil methane emissions have been found to vary depending on plant type and environmental conditions.

Also, PH-contaminated soil is seen increasingly as a considerable anthropogenic source of atmospheric methane. A recent in situ study of methane emissions in oil fields showed that methane emissions from contaminated soil were much higher (60-1,800 μg/m^-2^/h^-1^) than those from uncontaminated soil (29-33 μg/m^-2^/h^-1^) and suggested that PH biodegradation is mainly attributed to anaerobic microbial hydrocarbon degradation through methanogenesis [8]. Methane is a major greenhouse gas estimated to have a global warming potential (GWP) of 28–36 over 100 years [9]. When soils become contaminated with high oil concentrations, oxygen in the soil could be rapidly consumed, thereby resulting in anaerobic conditions [10]. This phenomenon can stimulate the syntrophy between PH-degrading fermentation bacteria and methanogenic bacteria, which increases soil methane emissions [11]. However, greenhouse gas emissions from soil are yet to be considered a major factor in rhizoremediation techniques and the effect of plants and soil amendments on the methane emission characteristics of rhizosphere microbial communities in petroleum-contaminated soil remains unclear.

In this study, the effects of major rhizoremediation factors (plants and soil amendments) on the remediation performance and potential methane emission characteristics in diesel-contaminated soil were investigated through an indoor pot experiment. Three types of soil systems, including a control condition (no planting), maize planting, and tall fescue planting, were set up. Maize and tall fescue were each selected as representative species of two plant groups: agronomic crops and grasses, respectively, which have distinct features for successful rhizoremediation, such as high biomass or extensive rhizosphere surface. Compost was applied as a soil amendment to investigate its usefulness associated with the degradation activity increase of indigenous microorganisms and methane emission reduction in the diesel-contaminated soil. The effects of exogenous microorganisms were studied compared to chemical nutrient (control).

During a 95-day period, rhizosphere soils were collected and soil residual total petroleum hydrocarbon (TPH) concentrations and potential methane emissions were analyzed. To follow up overall soil microorganism activity during remediation, dehydrogenase activity was analyzed as this enzyme is closely related to the microbial organic matter degradation process [12]. The rhizosphere bacterial community dynamics and key functional genes were investigated through Illumina MiSeq sequencing and quantitative polymerase chain reaction (qPCR), respectively. The abundances of *alkB* (alkane monooxygenase gene) and *pmoA* (particulate methane monooxygenase gene) were estimated through the qPCR to comprehend the microbial TPH degradation and methane oxidation activity during the experiment.

The results of this study can be used to develop rhizoremediation strategies for methane emission mitigation during the remediation of diesel-contaminated soil.

## Materials and Methods

### Soil, Plants, and Soil Amendments Preparation

Potting soil mixture was purchased from a commercial provider (Simpol, Korea) and organic matter content, total nitrogen (T-N), ammonium nitrogen (NH_4_^+^-N) and total phosphorus (T-P) were measured as 41.38%, 0.450%, 2.58 mg/kg, and 1278.07 mg/kg, respectively. The soil mixture was contaminated with diesel to obtain a final concentration of 30,000 mg diesel/kg-soil. The diesel-contaminated soil was aged at 20°C without light for 5 days and manually mixed once a day during the aging process. A chemical nutrient and compost were then prepared as soil amendments. To prepare the chemical nutrient, 2.7 g of NH_2_CONH_2_, 3.4 g of (NH_4_)_2_HPO_4_, and 0.9 g of K_2_SO_4_ were added to 1 L of distilled water (2 g/l nitrogen; 0.8 g/l phosphate; and 0.4 g/l potassium). The compost, which was a mixture of pig manure with sawdust (6:4; v/v), was matured for 6 months (Korea). The organic matter content of the compost (T-N, NH_4_^+^-N, and T-P) was measured at 67.74%, 2.630%, 371.60 mg/kg, and 22834.52 mg/kg, respectively.

Maize (*Zea mays*) and tall fescue (*Festuca arundinacea*) were prepared as remediation plants. Maize seedlings were purchased from a commercial vendor (Danong, Korea) and the height of the seedlings ranged from 80 to 160 mm. Tall fescue was cultivated for 2 months in a garden located at the New Engineering Building, Ewha Womans University (37°56′69′′ N, 126°94′87′′ E). The height of the tall fescue at the beginning of the experiment ranged from 100 to 140 mm.

The initial pH of the soil mixture was 5.2 and the final pH after the experiment period was increased to the range of 5.8~6.6, depending on the type of plants and soil amendments.

### Pot Experiment Preparation

A 3×3 factorial design pot experiment was conducted to investigate the effects of the experimental plants (control (no planting), maize, and tall fescue) and soil amendments (chemical nutrient and compost) on diesel-contaminated soil rhizoremediation and methane production potential. Two kilograms of diesel-contaminated soil were placed in each experimental pot (W 550 mm × L 195 mm × H 150 mm) for the control and tall fescue soil systems, whereas 5 kg were added to the maize soil system pot (W 600 mm × L 400 mm × H 210 mm). The chemical nutrient preparation and compost were added to the diesel-contaminated soil to a final concentration of 80 ml/kg and 100 g/kg, respectively. Then, 4~6 maize seedlings and 20 tall fescue seedlings were planted per pot, respectively. Two pots were prepared per each set of experimental conditions and then cultivated for 95 days at an indoor greenhouse located in the Asan Engineering Building, Ewha Womans University (37°56′65′′ N, 126°94′85′′ E). The average ambient temperature during the experiment period ranged from 16.4 to 31.6°C. All pots were watered 2~3 times a week to keep the surface soil from drying.

### Soil Sampling and Pretreatment

Bulk soil sampling was conducted every 1~2 weeks during the experiment and plant roots remained undisturbed during sampling. Bulk soil was sampled from five random points in each pot and mixed homogeneously. Rhizosphere soil sampling was conducted at the end of the pot experiment. Rhizosphere soil firmly attached to each plant root was sampled by manual shaking and mixed homogeneously. Half of each soil sample was freeze-dried for residual TPH concentration analysis and soil DNA extraction (Bondiro Vacuum Freeze-dryer, IlShinBioBase, Korea). The remaining half of the soil samples was spread over a clean vinyl sheet and air-dried in an indoor laboratory for approximately 24 hours. After pretreatment, all soil samples were kept at -4°C until further analyses; soil samples for DNA extraction were stored at -20°C.

### TPH Analysis

One gram of freeze-dried soil sample was placed in a glass test tube, after which 5 ml of hexane-acetone solution (1:1, v/v) was added to serve as an extraction solvent. Test tubes were tightly sealed using screw caps and Teflon tape to prevent solvent volatilization and then vortexed for 30 s. After 30 min of shaking (30°C, 200 rpm) and allowing the mixture to stand undisturbed for 30 min, 1 ml of supernatant was collected for each sample. A gas chromatography system (6980N Network GC System, Agilent Technologies, USA) equipped with a capillary column (L 30 m × ID 0.320 mm × T 0.25 μm; HP-5 GC column, J&W Scientific, Inc., USA) and a flame ionization detector (FID) was used to analyze the residual TPH concentration. The inlet and detector temperatures were 300°C and 320°C, respectively. N_2_ (99.999%, Dong-A Specialty Gases, Korea) was used as a makeup gas. The oven temperature was maintained at 60°C for the first 3 min, raised to 260°C at a 4°C/min rate, then raised to 310°C at an 84°C/min rate, and finally maintained for 5 min. A TPH standard curve was prepared using serial dilutions (500 to 40,000 ppm) of diesel fuel and FTRPH Calibration/Window Defining Standard (AccuStandard, Inc., USA).

### Dehydrogenase Activity Analysis

Dehydrogenase activity was analyzed as described by Bremner and Tabatabai [13]. Briefly, 0.5 g of air-dried soil sample was placed in a glass test tube, and 2 ml of Tris-HCl buffer (pH 7.6) and 1 ml of 1% (w/v) triphenyl tetrazolium chloride solution were added. The reaction mixture was incubated at 37°C in the dark for 24 h. Ten milliliters of ethanol (96%) was added as an extraction solvent, after which the mixture was vortexed for 30 s and centrifuged at 4,000 ×*g* for 5 min. The red color intensity of the supernatant was analyzed at 485 nm using a UV/Vis spectrophotometer (Libra S22 UV/Vis Spectrophotometer, Biochrom Ltd., UK). Dehydrogenase activity was expressed as the mass of triphenyl formazan produced by 1 g of dry soil during 24 h of incubation (μg/g).

### Potential Methane Emission (PME) Analysis

To estimate the methane emission potential of rhizosphere soil, a modified version of the GHG (greenhouse gas) incubation method was applied [14]. One gram of each air-dried soil sample was placed in a 35-ml glass vial. The moisture content of the soil samples was adjusted to field capacity (-33 kPa) by adding deionized water. The vials were then sealed with butyl stoppers and aluminum caps and incubated at 30°C for 60 days in quadruplicate. To measure accumulated CH_4_ concentrations, gas samples (100 μl) were collected 30 and 60 days post-incubation using a 300-μl gas-tight syringe (Hamilton, USA). Gas samples were analyzed with a gas chromatography system (7890A GC System, Agilent Technologies) equipped with a capillary column (L 30 m × ID 0.320 mm × T 1.80 μm; DB-624 GC Column, J&W Scientific, Inc., USA) and a flame ionization detector (FID). The inlet, oven, and detector temperatures were 230°C, 100°C, and 230°C, respectively, and N_2_ (99.999%, Dong-A Specialty Gases, Korea) was used as a makeup gas. A methane standard curve was prepared using serial dilutions (500 to 10,000 ppm) of methane gas (99%; Seoul Specialty Gases Co., Ltd., Korea).

Potential methane emissions were calculated with the following equation:

Eq. (1) $$\mathrm{PME}=\frac{x}{\mathrm{dry}\mathrm{soil}}\times \mathrm{MW}\times \chi \times \frac{P}{\mathrm{RT}}\times \frac{V_{T}}{10^{6}}$$

where, PME is the potential methane emission (μg/g-dry soil), x is the maximum methane concentration of headspace during the 60-day incubation period (ppmv), dry soil is the dry weight of the soil sample in the vial (g), MW is the molecular weight of the methane gas (g/mol), χ is the ratio of the molar mass of C to the molecular weight of the methane gas, P is the atmospheric pressure (atm), R is a gas constant, and V_T_ is the vial headspace volume (ml).

### DNA Extraction and Illumina MiSeq

Genomic DNA was extracted from 0.1 g of rhizosphere soil using a Nucleo Spin Soil Kit (Macherey-Nagel GmbH & Co. KG, Germany) and a BeadBeater-1 system (Biospec, USA). DNA extraction was performed following the manufacturer’s instructions. The DNA samples were collected in 50 μl of elution buffer and quantified using a SpectraMax QuickDrop Micro-Volume Spectrophotometer (Molecular Devices, USA). Extracted DNA samples were stored at -20°C until used.

A next-generation sequencing (NGS) approach was used to characterize bacterial community via the Illumina paired-end MiSeq sequencing platform (Macrogen Inc., Korea). The extracted DNA was used as a template for 16S sequencing library preparation, and the overall PCR process was conducted as described in a previous study [15]. Each composite primer was designed based on the 515f and 806r primer sequences, which amplify the V4 region of the microbial 16S ribosomal RNA gene. Operational taxonomic unit (OTU) clustering was performed using the CD-HIT-OTU program [16]. Raw reads with ambiguous bases and reads that didn’t match 515f/806r primers were removed. The median length was calculated for all reads and longer or shorter reads were trimmed through the length filter (200 bp ≤ good sequences ≤ 400 bp). The filtered reads were then clustered into clusters of duplicates if they were aligned at 5’ and shared 100% identity over the full length of shorter sequences, as reads are different in length. Chimeric reads were identified in this step and removed. Singletons were also removed in the OTU clustering process. OTUs were determined at 3% dissimilarity using QIIME software (Macrogen Inc.). The final taxonomy proportions and alpha diversity indices were calculated after normalized read number in each sample.

### Functional Gene qPCR

TPH-degrading bacteria and methane-oxidizing bacteria in the rhizosphere soil during rhizoremediation were estimated via qPCR using a CFX96 TouchTM Real-Time PCR Detection system (Bio-Rad Laboratories Inc., USA). Soil DNA samples used for Illumina MiSeq high-throughput sequencing were also analyzed via qPCR. The *alkB* and *pmoA* genes were amplified using the alkB-1F/alkB-1R, and A189f/mb661r primer pairs, respectively (Table S1) [17-19]. The *pmoA* standard curve was prepared via serial dilutions (10^3^ to 10^7^ gene copies) of linearized plasmids, each containing the respective cloned gene from Methylobacter luteus (NCIMB11914) [20]. qPCR was conducted for the *alkB* gene to estimate the relative abundance of TPH-degrading bacteria in the sample compared to the initial diesel contaminated soil (day 0).

The composition of the reaction mixture for quantitative PCR was as follows: 10x PCR buffer with MgCl_2_ (Genenmed Inc., Korea), 1x; dNTP mixture (Genenmed Inc.), 200 μM; forward primer, 0.2 μM; reverse primer, 0.2 μM; SYBR (Invitrogen, USA), 2x; Rox reference dye (Invitrogen), 2x; Taq polymerase (Genenmed Inc.), 0.025 U/μl. Two microliters of DNA was added as a template and the final volume was adjusted to 25 μl. qPCR was performed in duplicate. Then, 16S rRNA and *pmoA* gene abundance was calculated as copy numbers per gram of dry-soil, whereas *alkB* gene abundance was determined as the relative abundance to the initial value. Detailed information on target genes, primer sequences, and PCR conditions is provided in Table S1.

### Statistical Analysis

One-way and two-way analyses of variance (one-way ANOVA, two-way ANOVA) were conducted to compare the significant differences in the multiple data. The level of significance for different treatments was determined using a Scheffe post-hoc test at a 95% level (*p* < 0.05). Principal component analysis (PCA) was conducted to analyze the microbial community dynamics using CANOCO 4.5 software (Microcomputer Power, USA) and a biplot including major functional bacterial species was created. An analysis of similarities (ANOSIM) was also conducted to investigate the effects of each factor on the microbial communities. Pearson’s r was used in the correlation analysis to investigate the linear relationship between two data sets. SPSS Statistics Subscription Base Edition software (SPSS Inc., USA) and R (version 4.0.2) were used for most statistical analyses.

## Results

### Soil Microbial TPH Remediation Performance and Dehydrogenase Activity

**Fig. 1:**
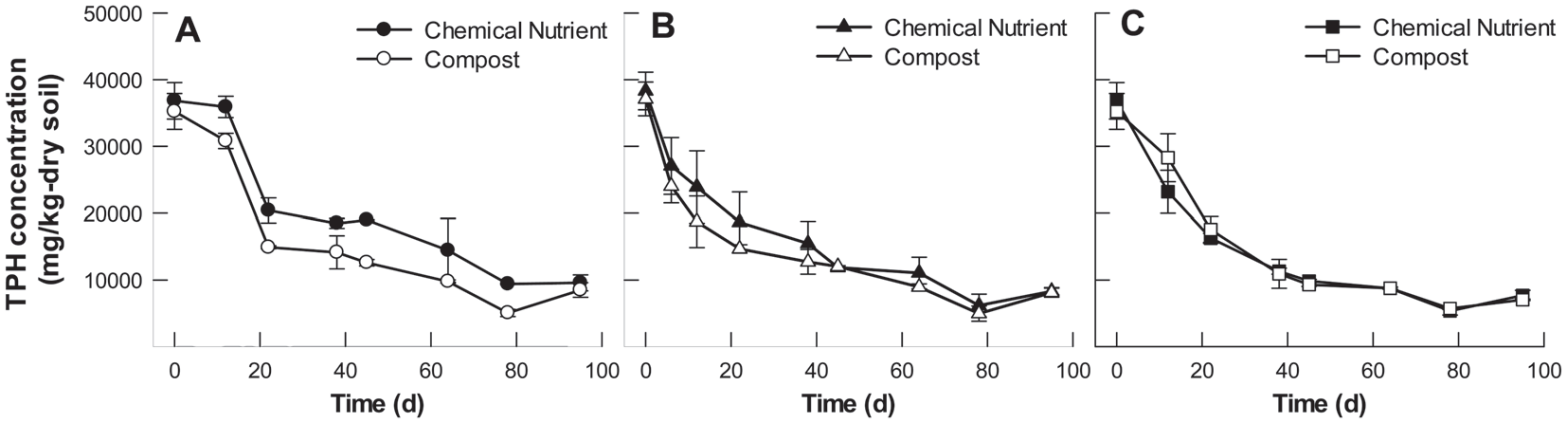
Time profiles of residual TPH concentration for each soil condition. (**A**) control (no planting), (**B**) maize planting, and (**C**) tall fescue planting.

Fig. 1 illustrates the changes in residual TPH concentrations in soil treated with different kinds of plant and soil amendments. The initial soil TPH contamination level was 36,696 ± 2,809 mg TPH/kg soil. In the control soil system, a lag phase was observed during the first 12 days, with no significant change in residual TPH concentration. TPHs then rapidly decreased until day 22, after which the concentration level (14,660~22,119 mg TPH/kg soil) was maintained until day 45. This level tended to gradually decrease after day 45 and the fastest TPH removal rate was observed when compost was added throughout the entire experimental period (Fig. 1A). In contrast, TPHs decreased immediately in soils treated with maize and tall fescue, and no lag phase was observed (Figs. 1B and 1C). In both conditions, the TPH concentration decreased rapidly to 11,438~12,054 and 8,996~10,003 mg TPH/kg soil, respectively, and TPH removal rates in the tall fescue-treated soil continued to decline after day 45.

**Fig. 2:**
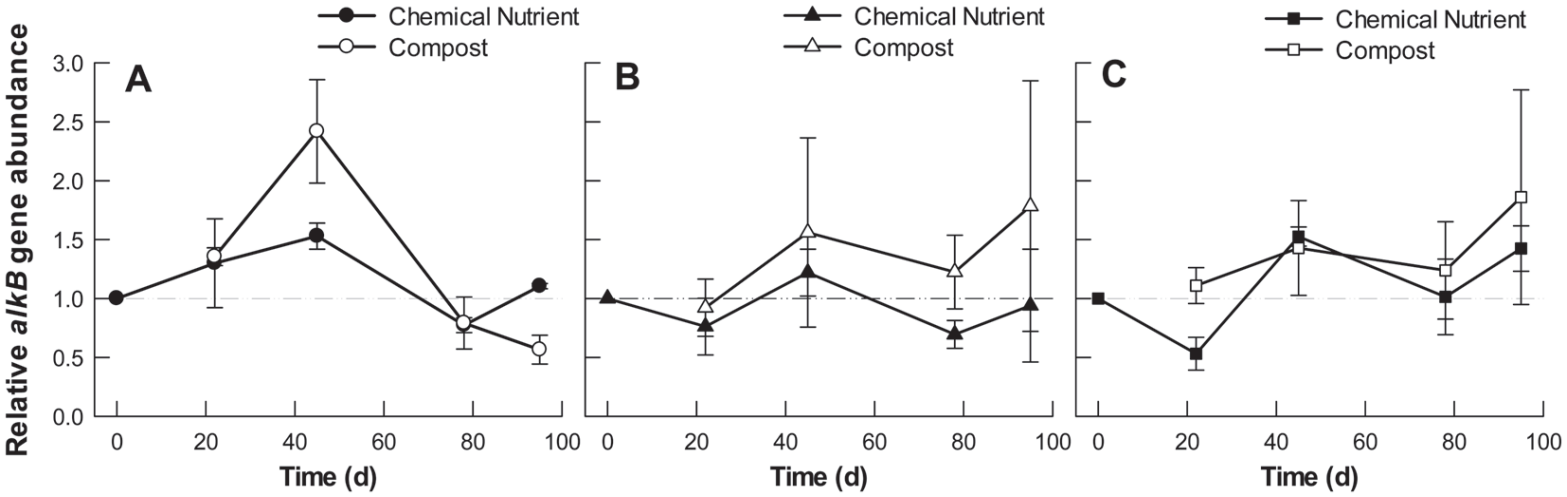
*alkB* gene abundance relative to the initial abundance at 0 d. (**A**) control (no planting), (**B**) maize planting, and (**C**) tall fescue planting.

Relative qPCR analysis was performed to investigate *alkB* gene dynamics associated with alkane degradation in each soil sample (Fig. 2). *alkB* gene abundance was used as an indicator of TPH-degrading bacteria abundance relative to the initial soil concentration. The relative *alkB* gene abundance in the control soil system on day 22 and 45 was higher than that of the initial soil, and then decreased. On day 45, the compost-amended maize soil system exhibited a 1.6-fold increase in *alkB* gene abundance compared to the initial soil concentration (Fig. 2B). Compared to the control, the soil systems treated with maize and tall fescue were found to maintain nearly the same abundance of TPH-degrading bacteria (relative to the beginning of the experiment) during the experimental period. Additionally, an increasing trend in relative *alkB* abundance was observed over time in compost-treated samples (Figs. 2B and 2C). According to our two-way ANOVA results, the type of plants and soil treatments had significant effects on TPH-degrading bacteria abundance only on day 22, which corresponded with the initial rhizoremediation stage (*p* < 0.05). On day 22, the average *alkB* gene abundance was high in the control system. However, no consistent results were observed in the remaining experimental time.

**Fig. 3:**
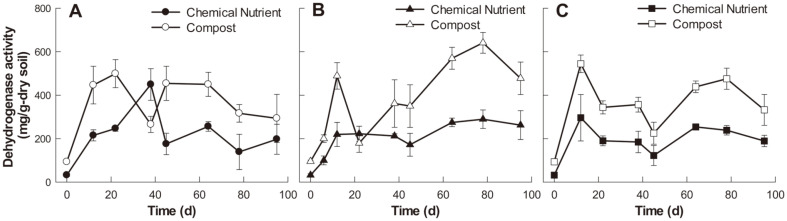
Dehydrogenase activity time profiles in the three studied soil conditions. (**A**) control (no planting), (**B**) maize planting, and (**C**) tall fescue planting.

Fig. 3 illustrates the changes in rhizosphere soil dehydrogenase activity treated with different kinds of plants and soil amendments. In the control soil system treated with the chemical nutrient preparation, dehydrogenase activity rapidly increased to 449 ± 73 μg TPF/(g dry soil/d) during the first 38 days (Fig. 3A), then decreased to 176± 49 μg-TPF/(g dry soil/d) on day 45, after which the activity level remained largely constant until the end of the experiment. In the case of compost addition, dehydrogenase activity increased up to 499 ± 65 μg TPF/(g dry soil/d) during the first 22 days and decreased after day 64. Furthermore, dehydrogenase activities increased up to 220 ± 56 and 296 ± 107 μg TPF/(g dry soil/d) until day 12 when the chemical nutrient preparation was added in maize- and tall fescue-treated soil, respectively. Both activity levels were maintained during the entire experiment. Moreover, in the soil system with maize and compost, dehydrogenase activity increased up to 489 ± 61 μg TPF/(g dry soil/d) on day 12 and showed a consistent increase after day 38. Similarly, when compost was added to the tall fescue soil system, the activity level increased up to 495 ± 117 μg TPF/(g dry soil/d) on day 12 and continued increasing steadily after day 68.

### Potential Soil Methane Emission and *pmoA* Gene Dynamics

**Fig. 4:**
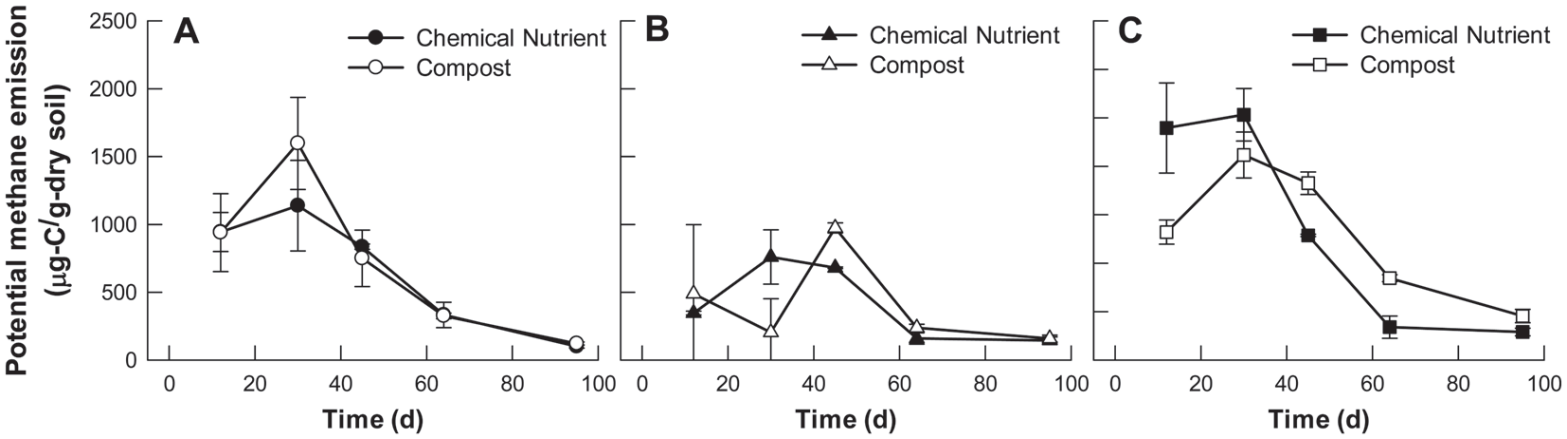
Potential methane emission in the three examined soil conditions during the experiment. (**A**) control (no planting), (**B**) maize planting, and (**C**) tall fescue planting.

Fig. 4 illustrates the methane emission potential of each soil system treated with different soil amendments at different experimental times. On day 12, the emitted methane level in the chemical nutrient-treated tall fescue system soil (958 ± 186 μg C/g dry soil) was 1.8-fold higher than that of compost-treated soil (528 ± 50 μg C/g dry soil) (Fig. 4C). On day 30, methane emissions in the soil systems were exhibited in the following order: control > tall fescue > maize, and the control soil treated with compost showed its highest methane emission of 1,597 ± 340 μg C/g dry soil (Fig. 4A). The maize soil system treated with compost temporarily showed its highest methane emission of 974 ± 37 μg C/g dry soil on day 45; however, it showed the lowest methane emissions among the soil systems overall (Fig. 4B). Potential methane emissions tended to decrease from day 64 onwards under all conditions examined herein.

Plant types were found to have a statistically significant effect on potential methane emission except for day 95 (*p* < 0.05). The maize soil system exhibited significantly lower average methane emissions than other soils during the entire experimental period as shown in Fig. 4B (*p* < 0.05). Soil amendment type also had statistically significant effects on methane emission after day 30, exhibiting interactive effects by plant type (*p* < 0.05). On day 30, the chemical nutrient-treated soil exhibited the highest methane emission, whereas methane emission levels after day 45 were higher in the compost-treated soil.

**Fig. 5:**
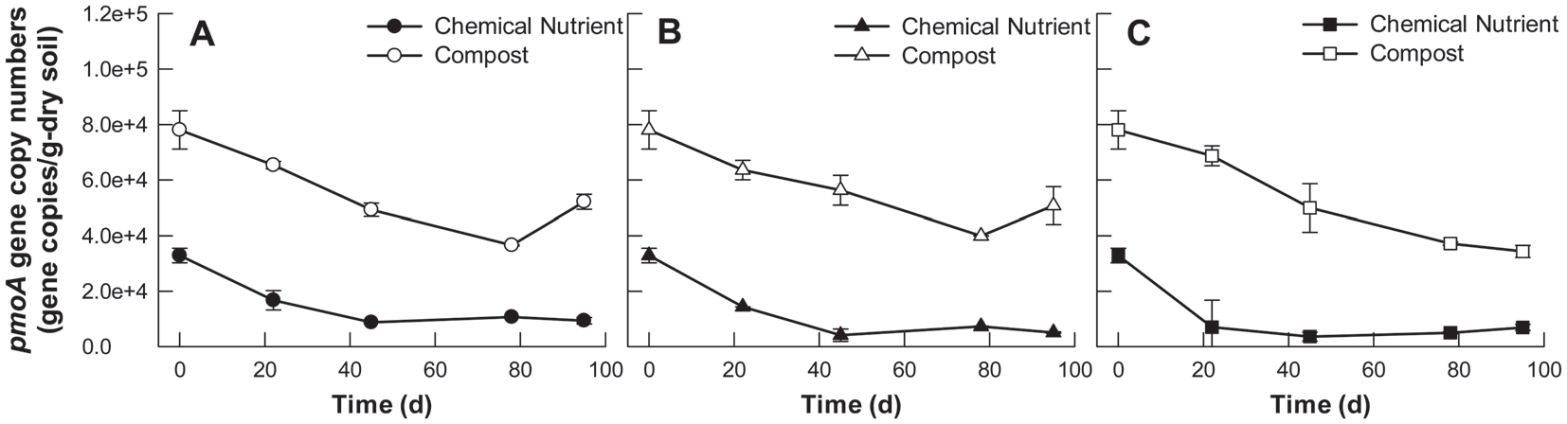
*pmoA* gene copy numbers in the three examined soil conditions during the experiment. (**A**) control (no planting), (**B**) maize planting, (**C**) tall fescue planting.

Absolute qPCR analysis was performed to investigate *pmoA* gene dynamics in each soil sample, which was used as an indicator of methane-oxidizing bacteria abundance (Fig. 5). In addition, time profiled of 16S rRNA gene are shown in Fig. S1. Across all plant type conditions, compost-amended soils exhibited higher *pmoA* gene copy numbers than chemical nutrient-treated soils. This suggests that several methanotrophs existed in the compost, which may support the results from Fig. 4. Specifically, relatively low levels of potential methane emission were observed in the compost-treated maize or tall fescue soil systems until day 30. According to the two-way ANOVA results, only soil amendment types were found to have statistically significant effects on *pmoA* gene copy numbers (*p* < 0.05). At the beginning of the experiment, the average *pmoA* gene copy numbers in the chemical nutrient- and compost-amended soils were 32,887 ± 2,169 and 78,059 ± 5,657 gene copies/g dry soil, respectively, and both exhibited a gradual decrease during the experimental period.

### Rhizosphere Bacterial Community Dynamics

Illumina MiSeq sequencing was performed to characterize the rhizosphere bacterial community in the soils treated with different kinds of plant and soil amendments. Table S2 summarizes the alpha diversities calculated from the sequencing data. Under all examined conditions, high bacterial diversities in rhizosphere soil samples were detected by the Shannon index (over 8.00) and inverse Simpson index (over 0.99) (Table S2).

Fig. S2 shows the profiles of bacterial community in each sample; any bacterial species that did not belong to the top 60 species were categorized as “others.” *Pseudoxanthomonas* was dominant in the initial contaminated soil (5.28~5.51%) but decreased under all conditions over time. Moreover, the relative abundance of *Nocardioides* and *Conyzicola* was higher in the control soil, whereas it decreased over time in the maize and tall fescue soil systems. Additionally, the relative abundances of *Marionbacter* in soil on day 95 were 4.44 ± 0.79 and 2.67 ± 0.93% in the maize and tall fescue soil systems, respectively, which were higher than that in the control soil (0.86 ± 0.49%). *Immundibacter* was identified in the initial contaminated soil at a low level of 0.09 ± 0.02% on average but tended to increase over time under all conditions. Similarly, the relative abundance of *Acidibacter* showed an increasing tendency in the compost-treated soil and was the highest in the tall fescue soil system on day 95 (6.77 ± 0.00%). *Bradyrhizobium* showed a relative abundance of 1.14~2.68% over the entire experimental period under all conditions particularly on day 45, at which point it was higher in the maize and tall fescue soil systems (2.09 ± 0.26 and 2.07 ± 0.55%, respectively) than in the control soil (1.81 ± 0.14%). In addition to the aforementioned bacteria, *Paludibaculum, Blastochloris, Rhodoplanes, Rhizobacter, Terrimonas, Hyphomicrobium*, and *Thermoanaerbaculum* were found to be more abundant in the maize and tall fescue soil system than in the control soil.

**Fig. 6:**
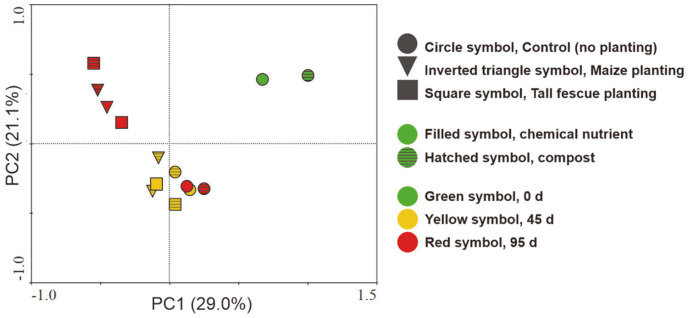
Principal component analysis (PCA) of soil bacterial community after 0 d, 45 d, and 95 d from the start of the experiment.

PCA was also performed to compare the bacterial communities in each soil system (Fig. 6). The bacterial community of the initial contaminated soils (day 0) was different depending on the type of soil amendments, which is likely due to the existence of various exogenous microorganisms in the compost. Significant changes were observed in overall bacterial community as a function of time. Control soil bacterial structures were relatively stable from day 45 until day 95, and that of chemical nutrient-treated soil, especially, showed the least change between the two aforementioned time points. On day 45, the chemical nutrient-treated maize and tall fescue soil systems showed similar bacterial structures, whereas the compost-treated soils in both systems were significantly different from each other. On day 95, the chemical nutrient- and compost-treated soil in the tall fescue system exhibited the highest similarity distances (*i.e.*, the most different structures) among the rhizoremediation conditions. The results of the ANOSIM test showing detailed information on the effects of each factor on the microbial communities are shown in Table 1. There was a significant difference in the bacterial communities based on the grouping of sampling time with the R-value of 0.6505 (*p* < 0.05). Plant type was demonstrated to be a major factor that contributed to the statistical difference in the bacterial communities. The increased R-value from 0.3542 (day 45) to 0.7569 (day 95) indicates that the dissimilarity of microbial community based on the grouping of plant type increased during the experiment (Table 1). The R-value comparing the bacterial communities with or without compost addition was 0.5818 on day 45 and decreased during the experiment, showing no significant difference among them on day 95.

## Discussion

### Effect of Rhizoremediation Factors on TPH Removal Performance and Dehydrogenase Activity

Plants that promote the decomposition of organic contaminants in soil share common characteristics, including extensive and fibrous roots that form an extended rhizosphere [21]. These plants include many common grasses, maize, and legumes (*e.g.*, soybeans, peas, beans) and have been assessed in many TPH-contaminated soil rhizoremediation studies [22-24]. In this study, maize and tall fescue were examined in an indoor pot experiment, and both plant species were found to significantly improve TPH removal rates (Fig. 1). A previous study reported that TPH removal efficiencies in soils planted with ryegrass (*Festuca perennis*), sorghum (*Sorghum bicolor*), maize, alfalfa (*Medicago sativa*), bermudagrass (*Cynodon dactylon*), rice (*Oryza sativa*), kudzu vine (*Pueraria montana*), and Spanish needles (*Bidens bipinnata*) were significantly higher than in the control soil [25].

The *alkB* gene, which is used as an indicator of TPH-degrading bacteria abundance, maintained its initial gene copy number levels during the entire experimental period in the maize or tall fescue soil systems (Figs. 2B and 2C). Particularly, compost-amended soil exhibited a higher *alkB* gene abundance on day 95 than that at the initial rhizoremediation stage. Moreover, the PCA analysis results showed that overall bacterial community in soil systems was significantly changed during rhizoremediation (Fig. 6). To maintain ecosystem homeostasis, microbial composition can change in different ways in response to environmental disturbances (*e.g.*, oil spills) depending on community characteristics such as resistance, resilience, and functional redundancy [26]. Particularly, microbial communities with functional redundancy can perform like the original community despite microbial composition alterations [26]. In this study, although the soil microbial structure changed over time after diesel contamination, the microbial TPH degradation performance (*i.e.*, *alkB* gene relative abundance) was largely preserved in the soil systems with plants and compost. Therefore, the functional redundancy of the soil systems during rhizoremediation was attributed to plant (maize and tall fescue) and compost introduction.

In the previous study of petroleum contaminant rhizoremediation, dehydrogenase activity was demonstrated to be more relevant in TPH removal efficiency than the TPH-degrading microorganism biomass itself [27]. In this study, the average rate of change in dehydrogenase and the average removal rate of TPH in each sampling time interval showed a moderate positive linear relationship with the Pearson correlation coefficient of 0.324 (*p* < 0.1).

Since microbial communities in the petroleum-contaminated soils have low biodiversity and biomass, it is hard to maintain favorable environmental conditions for the survival of remediation plants [4]. Soil amendments can be applied for the purpose of increasing the rate or extent of biodegradation of PHs, which improves the activity of the soil microorganisms. In this study, the potting soil mixture was contaminated with a high concentration of diesel (30,000 mg diesel/kg soil). As the indigenous microorganisms in the soil mixture may not be capable of degrading the high loads and wide ranges of substrates in diesel, compost and chemical nutrient (control) were added to study the effect of soil amendments on the enhancement of the overall degrading activity of the soil microorganisms. When compost is added to petroleum-contaminated soil, exogenous microorganisms including bacteria and fungi are introduced into the soil ecosystem, which stimulates the degradation of various organic contaminants into less toxic substances. Moreover, high nutrient loads in compost can enhance soil fertility and plant growth, resulting in the removal of organic contaminants in the soil [28]. A previous study reported that compost addition increased diesel removal from soils both with and without ryegrass while the soil with ryegrass showed a much lower level of residual diesel concentration [29]. Likewise, in this study, the TPH removal efficiency and dehydrogenase activity of the compost-amended soil with maize and tall fescue planting were higher than those of other soils.

### Effects of Rhizoremediation Factors on Potential Methane Emission 

Plants affect soil methane emission by secreting various organic acids as plant root exudation, which can be utilized by microorganisms that participate in the production and elimination of methane [30]. Moreover, many previous studies have reported that rice roots can reduce soil methane emissions in paddy fields. Plant roots enable the ambient oxygen to enter the rhizosphere, thereby changing the soil redox potential [31]. This leads to the retention of a certain portion of the soil-emitted methane, resulting in favorable conditions for methanotrophs [32]. This study found that plant types had statistically significant effects on potential methane emission and the maize planting soil system showed the lowest methane emission among the soil systems (Fig. 4). However, no significant difference in *pmoA* gene copy numbers (*i.e.*, an indicator of methanotroph abundance in the microbial community) was observed (Fig. 5).

**Fig. 7:**
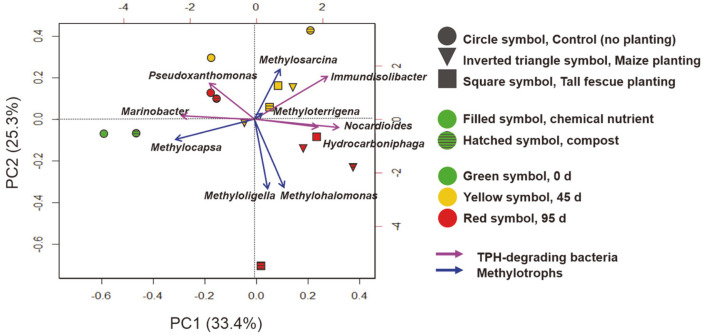
Multivariate analysis including 10 major functional species of TPH-degrading bacteria and methylotrophs. PCA biplot represents the bacterial community in different soil systems. Arrows represent projections of the species that are responsible for the differences between groups.

Many field studies of maize soil (usually conducted in crop rotation systems with legumes due to their having several advantages) demonstrated that the plots showed negligible methane flux, or they acted as a methane sink [33-36]. Meanwhile, the maize rhizosphere soils in the previous study were demonstrated to have completely different microbial community and methanogenic bacterial species compared with those of the rice rhizosphere soils. [37]. Another maize field study with biochar applications showed a significant decrease in the soil of saprotrophic fungi, which were previously reported to produce methane without the involvement of methanogenic archaea [38, 39]. Similarly, in our study, we found that the maize soil showed lower methane emissions especially with compost, and the composition of microbial community significantly altered during the rhizoremediation. Thus, it is assumed that the maize and compost altered the major composition of methanotrophic species (Fig. 7), resulting in less methane emission in the TPH-contaminated environment. However, further testing is needed with greater consideration given to methanogenesis and mcrA gene copy numbers.

The results in Figs. 4 and 5 confirmed that the addition of compost both increased the potential methane emission and the *pmoA* gene copy numbers in the soil. Organic fertilizer application can improve soil quality, but also increases methane emissions by increasing the nutrients and organic acid contents in the soil, which can then be utilized by methanogens [40]. On the other hand, compost is a rich source of useful microorganisms, including a large number of methanotrophs [41]. A previous study determined that soil amended with organic fertilizer exhibited a 3-fold increase in methane oxidation rate compared to soils receiving mineral fertilizers, with significant enhancement in methanotroph abundance in organically fertilized soil [42]. Meanwhile, an apparent decrease in potential methane emissions over time was observed under all examined conditions, which may be due to a decrease in the amount of organic matter available for soil microorganisms to utilize as a carbon source.

### Major Functional Bacterial Species During Rhizoremediation

Based on our indoor pot experiment bacterial community analysis, various microorganisms associated with TPH degradation were identified (Fig. S2). *Pseudoxanthomonas* is known to be able to degrade diesel, pyrene, phenanthrene, and BTEX (Benzene, Toluene, Ethylbenzene, Xylene) compounds [43]. However, the relative abundance of *Pseudoxanthomonas* was only found to be high at the initial stage of the experiment and tended to decrease over time, indicating that the contribution of this genus to TPH removal capacity during the rhizoremediation was likely negligible. *Nocardioides*, which occurred at relatively high proportions in the control soil, has been reported to degrade various petroleum products [44], and was therefore likely a major TPH-degrading bacterial genus in the control soil system. *Marinobacter*, which occurred at a high relative abundance in the maize and tall fescue soil systems on day 95, is also a bacterial genus largely known for its TPH-degrading capacity, as reported in *M. hydrocarbonoclasticus* and *M. nanhaiticus* [45-46]. Therefore, we concluded that the *Marinobacter* genus was likely the main contributor to TPH degradation in soil systems with plants. *Immundisolibacter*, which showed low relative abundance at the beginning and gradually increased over time, has been reported to degrade polyaromatic hydrocarbons (PAHs) [47]. Up to 75% of diesel fuel is composed of alkane compounds with the chemical equation C_n_H_2n+2_. However, polyaromatic hydrocarbon compounds such as naphthalene and alkylbenzene also occur in diesel fuel. Therefore, *Immundisolibacter* may have participated in PAH degradation during the rhizoremediation of diesel-contaminated soil.

Notably, this study identified various microorganisms related to methane oxidation; however, not all of them were included in the top sixty bacterial community species. For instance, methylotrophs such as *Methylohalomonas, Methylocapsa, Methyloligella, Methyloterrigena*, and *Methylosarcina* were observed at relative abundances of 0.01~1.75% through the entire experimental period. Methylotrophs are groups of microbes that can use one-carbon compounds such as methanol or methane as energy sources [48]. Among them, *Methylocapsa* and *Methylosarcina* have been reported to have methane-oxidizing capacity via the *pmoA* gene [49, 50]. Therefore, it was assumed that the aforementioned methylotrophs affected methane emission characteristics during rhizoremediation, including potential methane emission and *pmoA* gene copy numbers (Figs. 4 and 5).

Multivariate analysis including 10 major functional bacterial species was performed and represented as a PCA-biplot of microbial structures of the soil systems (Fig. 7). The 0 d soil systems were clearly characterized with the high abundance of *Marinobacter* and *Methylocapsa*. Most of the microbial structures on day 45 showed high abundance of *Immundisolibacter* and Methylosarcina, while the control soil without compost had a high abundance of Pseudoxanthomonas. On day 95, most of the soil systems with planting showed a high abundance of *Nocardioides* and Hydrocaboniphaga, while the maize planting soil with compost showed a high abundance of Methyloligella and Methylohalomonas. The control soil on day 95 was detected to have a clear difference compared to the planting soil with high abundances of *Pseudoxanthomonas* and *Marinobacter*. Therefore, we concluded that not only the overall microbial structures (Fig. 6), but also the major functional bacterial species that contributed to the TPH degradation and methane oxidation changed in each soil system during the experiment (Fig. 7). Notably, the maize planting soil with compost was distinguished by high abundance of the two methylotrophs (*Methyloligella* and *Methylohalomonas*). Similarly, in the previous study, distinct methylotrophic and methanogenic species were detected in the maize soil rhizosphere compared to other plant rhizospheres [37].

## Conclusions

This study investigated the effects of plants and soil amendments on methane emission characteristics during the rhizoremediation of diesel-contaminated soil. Based on TPH removal efficiency and greenhouse gas emission reduction, maize and tall fescue soil planting was found to be an effective remediation enhancement strategy. Moreover, compost was found to be an effective soil amendment, improving overall remediation efficiencies while also increasing both potential methane emissions and *pmoA* gene abundance in soils. A high level of diesel pollution could stimulate the synergism between plants and rhizobacteria in response to environmental stress, and therefore this synergism could be utilized as a source of various useful functional microbial resources, thereby contributing to TPH removal and methane emission reduction. In conclusion, this study suggests that greenhouse gas emission should be considered a major factor in rhizoremediation studies for the development of sustainable biological remediation technologies in response to global climate change.

## Supplemental Material

Supplementary data for this paper are available on-line only at http://jmb.or.kr.

## Figures and Tables

**Table 1:** ANOSIM table of bacterial community in soil samples by the grouping of sampling time (day 0, 45, 95), plant type (none, maize, tall fescue), and soil amendment type (chemical nutrient, compost).

Day (d)	Time	0	45		95	

Factor		Compost	Plant	Compost	Plant	Compost
R-value	0.6505	1	0.3542	0.5818	0.7569	0.0389
*p*	<0.0001	0.33333	0.0206	0.0038	<0.0001	0.2794
