# Supplementary material for: Effects of Plant and Soil Amendment on Remediation Performance and Methane Mitigation in Petroleum-Contaminated Soil
Source: J Microbiol Biotechnol. 2020 Oct 23;31(1):104–14. doi: 10.4014/jmb.2006.06023 (PMC9705697; doi:10.4014/jmb.2006.06023)
Supplement: Supplementary file 1 [file jmb-31-1-104-supple.pdf]

https://doi.org/10.4014/jmb.2006.06023

Table S1. Primer details and qPCR condition for functional gene quantification

Functional gene	Coding enzyme	Primer	Sequence (5’-3’)	qPCR condition	Ref.
**16S rRNA**	16S rRNA sequence of *Escherichia coli*	340F<br>805R	TCC TAC GGG AGG CAG CAG<br>GAC TAC HVG GGT ATC TAA TCC	94 °C 3 min;<br>94 °C 30 s, 50 °C 30 s, 72 °C 30 s, 82 °C 30 s	[17]
alkB	Rubredoxin dependent alkane monooxygenase	alkB-1F<br>alkB-1R	AAYACNGCNCAYGARCTNGGNCAYAA<br>GCRTGRTGRTCNGARTGNCGYTG	95 °C 2 min;<br>95 °C 20 s, 55 °C 20 s, 72 °C 40 s, 82 °C 30 s	[18]
pmoA	Alpha subunit of particulate methane monooxygenase	A189f<br>mb661r	GGN GAC TGG GAC TTC TGG<br>CCG GMG CAA CGT CYT TAC C	95 °C 3 min;<br>95 °C 15 s, 63 °C 30 s, 72 °C 30 s, 82 °C 30 s	[19]

Table S2. Number of OTUs and alpha-diversity indices for rhizosphere bacterial communities after 0, 45, and 95 d.

Plant	Soil amendment	Time	No. of OTUs	Chao1[a]	Shannon[b]	Good's Coverage[c]
Control	Chemical nutrient	0 d	1267	1702.41	8.02	0.99
		45 d	1458	1913.84	8.16	0.99
		95 d	1530	1991.21	8.33	0.99
	Compost	0 d	1388	1792.64	8.26	0.99
		45 d	1654	2115.88	8.63	0.99
		95 d	1516	1995.73	8.36	0.99
Maize	Chemical nutrient	0 d	1267	1702.41	8.02	0.99
		45 d	1681	2204.99	8.31	0.98
		95 d	1793	2337.45	8.69	0.98
	Compost	0 d	1388	1792.64	8.26	0.99
		45 d	1785	2269.48	8.65	0.98
		95 d	1811	2359.67	8.69	0.98
Tall fescue	Chemical nutrient	0 d	1267	1702.41	8.02	0.99
		45 d	1725	2269.34	8.39	0.98
		95 d	1689	2235.95	8.58	0.98
	Compost	0 d	1388	1792.64	8.26	0.99
		45 d	1646	2134.91	8.47	0.98
		95 d	1699	2199.02	8.62	0.98

[a] Chao1 is an index used to evaluate the overall bacterial population richness.
[b] Shannon is an index used to evaluate the diversity within the bacterial population.
[c] Good’s coverage is calculated as C=1-(s/n), where s is the number of unique OTUs and n is the number of individuals in the sample. This index provides a relative measure of how well the sample represents the overall environment.

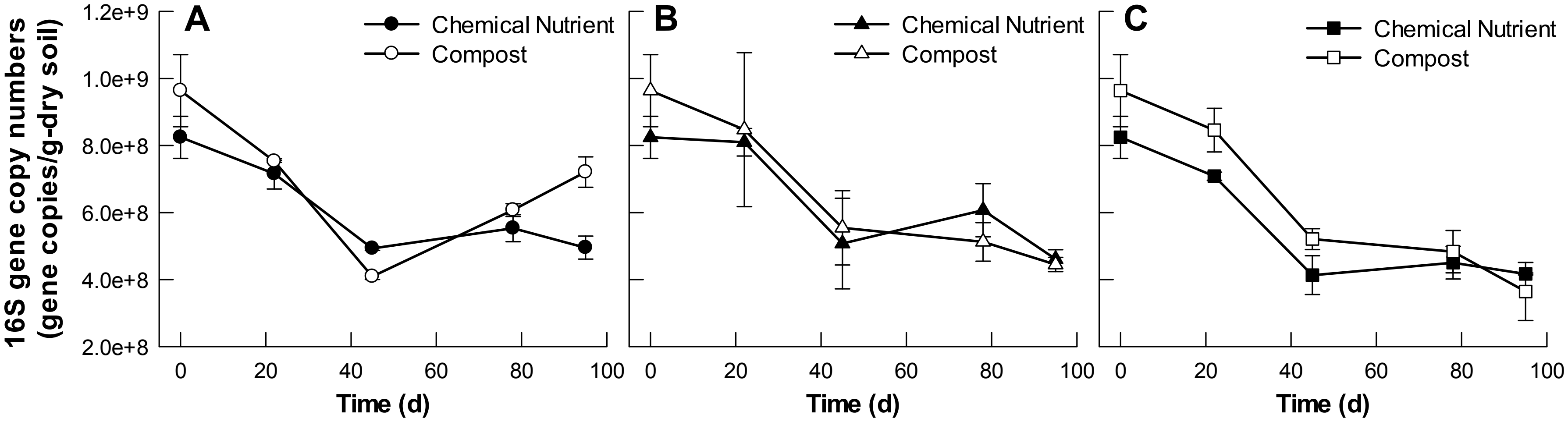

Fig. S1. 16S rRNA gene copy numbers in the three studied soil conditions. (**A**) control (no planting), (**B**) maize planting, and (**C**) tall fescue planting.

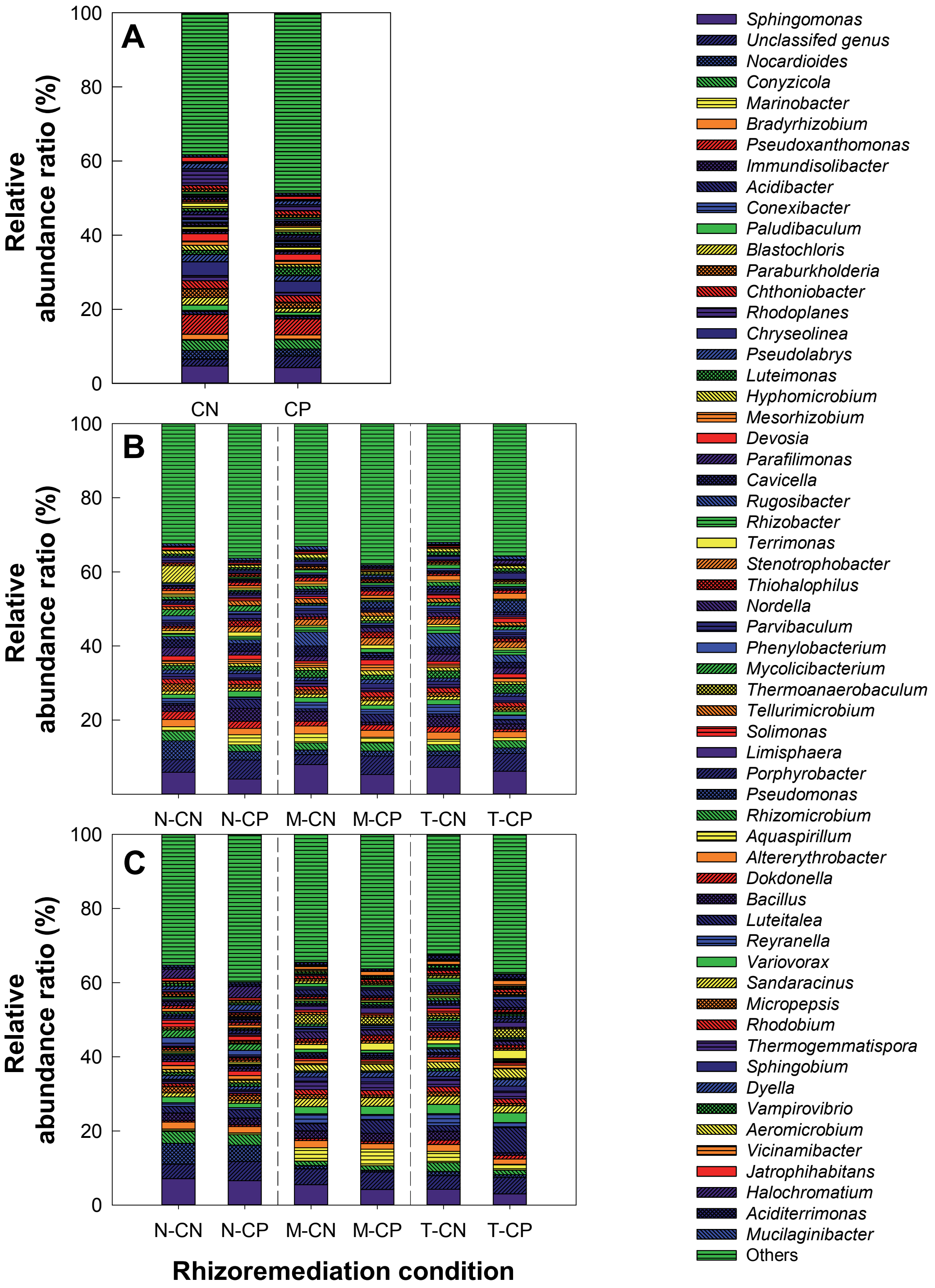

Fig. S2. Genus-level comparisons of rhizosphere bacterial community structures after (A) 0 d, (B) 45 d, and (C) 95 d from the start of the experiment. N, control (no planting); M, maize planting; T, tall fescue planting; CN, chemical nutrient addition; CP, compost addition.
